# An unbiased Hessian representation for Monte Carlo PDFs

**DOI:** 10.1140/epjc/s10052-015-3590-7

**Published:** 2015-08-12

**Authors:** Stefano Carrazza, Stefano Forte, Zahari Kassabov, José Ignacio Latorre, Juan Rojo

**Affiliations:** TIF Lab, Dipartimento di Fisica, Università di Milano and INFN, Sezione di Milano, Via Celoria 16, 20133 Milan, Italy; Dipartimento di Fisica, Università di Torino and INFN, Sezione di Torino, Via Pietro Giuria 1, 10125 Turin, Italy; Departament d’Estructura i Constituents de la Matèria, Universitat de Barcelona, Diagonal 647, 08028 Barcelona, Spain; Rudolf Peierls Centre for Theoretical Physics, University of Oxford, 1 Keble Road, Oxford, OX1 3NP UK

## Abstract

We develop a methodology for the construction of a Hessian representation of Monte Carlo sets of parton distributions, based on the use of a subset of the Monte Carlo PDF replicas as an unbiased linear basis, and of a genetic algorithm for the determination of the optimal basis. We validate the methodology by first showing that it faithfully reproduces a native Monte Carlo PDF set (NNPDF3.0), and then, that if applied to Hessian PDF set (MMHT14) which was transformed into a Monte Carlo set, it gives back the starting PDFs with minimal information loss. We then show that, when applied to a large Monte Carlo PDF set obtained as combination of several underlying sets, the methodology leads to a Hessian representation in terms of a rather smaller set of parameters (MC-H PDFs), thereby providing an alternative implementation of the recently suggested Meta-PDF idea and a Hessian version of the recently suggested PDF compression algorithm (CMC-PDFs). The mc2hessian conversion code is made publicly available together with (through LHAPDF6) a Hessian representations of the NNPDF3.0 set, and the MC-H PDF set.

## Introduction

The reliable treatment of uncertainties on the Parton Distributions (PDFs) of the proton is currently an essential ingredient for LHC phenomenology (see for example Refs. [1–5] for recent reviews). PDF uncertainties are of a peculiar nature, because they are uncertainties on a space of functions, and two main methods have been used to provide a representation of them: the Hessian method and the Monte Carlo (MC) method.

In the Hessian method (currently used for instance in the MMHT14 [6] and CT10 [7] PDF sets), a parametrization based on a fixed functional form is introduced, and a multigaussian probability distribution is assumed in the space of parameters. Uncertainties are then given as the inverse of the covariance matrix of this multigaussian distribution. This is usually obtained, assuming linear error propagation and the least-squares method, as the Hessian matrix with respect to the parameters of a figure of merit ($\chi ^{2}$) at its minimum, which is viewed as the best-fit PDF. In the Monte Carlo method (currently used for instance in the NNPDF3.0 [8] PDF set) PDFs are delivered as an ensemble of replicas which provide a discrete (Monte Carlo) representation of the underlying probability distribution: uncertainties are then simply obtained as moments of this probability distribution.

The Monte Carlo method has the twofold advantage that no Gaussian and linear error propagation assumption is necessary, and also, that PDFs can then parametrized with a general-purpose functional form with a large number of parameters (such as neural networks), for which the least-squares method would fail. The Hessian method, on the other hand, has the advantage that the (orthogonal) eigenvectors of the Hessian matrix may be treated as nuisance parameters. This is often a desirable feature when PDFs are used in experimental analysis, because other sources of uncertainty are also represented as nuisance parameters, and also because through standard methods [9] it is then possible to the determine a subset of nuisance parameters which is most important for a given cross-section or distribution.

Whereas deviations from Gaussianity may be important in specific kinematic regions, especially when limited experimental measurements are available and PDF uncertainties are driven by theoretical constraints (such as for example the large-*x* region, relevant for new physics searches), in most cases, and specifically when PDF uncertainties are small and driven by abundant experimental data, the Gaussian approximation is reasonably accurate. This then raises the question of whether in such case, in which everything is Gaussian and the Hessian approximation is adequate, one could have the best of possible worlds: a Hessian representation with the associate advantages, but without having to give up the use of a general-purpose flexible functional form.

It is the purpose of the present paper to achieve this goal. We will do this by using the MC replicas themselves as the basis of the linear representation of the original MC sample. Indeed, we will show that if replicas of a very large Monte Carlo set ($N_{\mathrm{{rep}}}=1000$ replicas) are represented as a linear combination of a subset of them, not only it is possible to achieve very good accuracy by using a much smaller subset of replicas as basis functions, but in fact there is an optimal number of basis replicas, in that the degeneracy of replicas is such that larger bases would no longer be linearly independent. It turns out that this optimal number is quite small, of the same order of magnitude as the typical number of Hessian eigenvectors for standard PDF sets such as MMHT14 or CT10. All this is true if the basis replicas are suitably chosen, which we do using a genetic algorithm. We can then simply construct a Hessian representation in the space of these linear expansion coefficients, with essentially no information loss or further bias introduced in comparison to the starting Monte Carlo representation. It is thus possible to provide a faithful, unbiased Hessian representation of any Monte Carlo PDF set, such as those provided by NNPDF.

It is interesting to observe that the inverse problem, namely the conversion of a Hessian PDF set into a Monte Carlo representation, has already been considered and solved [10]. An important advantage of being able to provide a MC representation of Hessian sets is the construction of combined PDF sets, which incorporate the information contained in several individual sets, as required for instance for Higgs boson coupling extraction or New Physics searches at the LHC. Currently, the recommended procedure (the so-called PDF4LHC recommendation [11, 12]) is to take an envelope, which has no clear statistical meaning. However, once converted into a Monte Carlo representation, PDFs based on a common dataset can be combined in a simple way [1, 3, 10, 13]. In this context, also the problem discussed in this work, namely the conversion from Monte Carlo to Hessian, has also been handled in the so-called “Meta-PDF” approach [13]. In this approach, a functional form similar to those used in the MMHT14 and CT analyses, the “meta-parametrization”, is fitted to the combined Monte Carlo PDF set. This clearly achieves the same goal as the conversion considered here, but with the further usual bias that a choice of functional form entails. If applied to a combined PDF set, the methodology presented here provides thus an unbiased alternative to the Meta-PDF method of Ref. [13].

The paper is organized as follows. In Sect. 2 we describe in the detail our methodology for the Monte Carlo to Hessian conversion. Then, in Sect. 3 we first, apply our methodology to a native Monte Carlo set, NNPDF3.0, benchmark its accuracy, and show that we end up with an optimal number of Hessian eigenvectors of order of a hundred. We then apply the methodology to a Monte Carlo set obtained by applying the Watt–Thorne [10] method to a starting Hessian PDF set, MMHT14. This provides a closure test of the methodology: we can check explicitly that the starting set is reproduced very well. Finally, in Sect. 4 we provide a Hessian representation of a Monte Carlo set obtained by combining several underlying PDF sets (either native Monte Carlo or converted to Monte Carlo from Hessian). We end up with a set of eigenvectors, the MC-H PDFs, which is of similar size of the compressed Monte Carlo PDF obtained recently [14] by applying compression algorithms to the large combined replica set, the so-called CMC-PDFs. Therefore, the PDF set which we obtain in this case provides an alternative to either the Meta-PDFs of Ref. [13], of which it provides an unbiased version, or to the CMC-PDFs of Ref. [14], of which it provides a Hessian version. Details of PDF delivery in LHAPDF6 are presented in Sect. 5, where conclusions are also drawn. In Appendix A we discuss an alternative strategy to construct a Hessian representation of MC sets, which is used to validate our main methodology, and might turn out to be advantageous for future applications.

## Methodology

As discussed in the introduction, the basic idea of our approach is to construct a linear representation for a set of Monte Carlo PDF replicas by expressing them as a linear combination of a small subset of them. Linearized error propagation, which is at the basis of the Hessian approach, can then be applied to the expansion coefficients, which immediately provide a representation of the Hessian matrix. It is important to observe that by “PDF replica” we mean the full set of PDFs at the parametrization scale, i.e., seven PDFs provided as a function of *x* for some fixed $Q^2$ value, denoted in the following by $Q_0^2$. These are all represented as a linear combination of the basis replicas with fixed coefficients, which thus do not depend on either the PDF or *x* value. Note that, because of the linearity of perturbative evolution, once a replica is expressed as a linear combination at the reference scale, all PDFs at all scales (including heavy flavors generated dynamically above the corresponding thresholds) are then given by the same linear combination of basis replicas. This in particular ensures that sum rules are automatically satisfied.

We will first, describe how the Hessian matrix is constructed, and then, the optimization of parameters that characterize the procedure, specifically the choice of basis replicas.

### Construction of the Hessian matrix

We start assuming that we are given a prior set of PDFs represented as MC replicas $\{ f^{(k)}_{\alpha }\}_{k=1,\ldots ,N_{\mathrm{{rep}}}}$ where $\alpha =1,\ldots ,N_{\mathrm{{pdf}}}$ denotes the type of PDF, i.e. $N_\mathrm{{pdf}}=2N_f+1$: $N_f$ quarks and antiquarks and the gluon. In order to simplify the notation, we drop the explicit dependence of the PDFs on *x* and $Q^2$. The central idea of our strategy consists of finding a subset of replicas, denoted by $\{ \eta ^{(i)}_{\alpha } \}_{i=1,\ldots ,N_{\mathrm{{eig}}}} \subset \{f^{(k)}_{\alpha }\}$, such that any replica of the prior set, $f^{(k)}_{\alpha }$, can be represented as a linear combination

1 $$\begin{aligned} f_{\alpha }^{(k)}\approx & {} f^{(k)}_{H,\alpha } \equiv f^{(0)}_{\alpha } + \sum _{i=1}^{N_{\mathrm{{eig}}}} a_i^{(k)} (\eta ^{(i)}_{\alpha } - f^{(0)}_{\alpha }),\nonumber \\&\quad k=1,\ldots ,N_{\mathrm{{rep}}}, \end{aligned}$$

where $f^{(0)}_{\alpha }$ is the central (average) value of the prior MC set; $a_i^{(k)}$ are constant coefficients, independent of $\alpha ,x$ and $Q^{2}$; and $f^{(k)}_{H,\alpha }$ denotes the new Hessian representation of the original replica $f_{\alpha }^{(k)}$. Note that by construction the central value of the Hessian representation is the same as that of the original MC set.

In order to determine the parameters $\{a_i^{(k)}\}$ we first define the covariance matrix in the space of PDFs for the prior set of replicas as

2 $$\begin{aligned} \mathrm{cov}^{\mathrm{{pdf}}}_{ij,\alpha \beta }\equiv & {} {{N_{\mathrm{{rep}}}}\over {N_{\mathrm{{rep}}} -1}}(\langle f^{(k)}_{\alpha }(x_i,Q^2_0)\cdot f^{(k)}_{\beta }(x_j,Q^2_0)\rangle _{\mathrm{{rep}}}\nonumber \\&-\langle f^{(k)}_{\alpha }(x_i,Q^2_0) \rangle _{\mathrm{{rep}}} \langle f^{(k)}_{\beta }(x_j,Q^2_0)\rangle _{\mathrm{{rep}}}), \end{aligned}$$

where the averages are performed over the original set of $N_{\mathrm{{rep}}}$ replicas. Then, we construct a figure of merit, $\chi ^{2(k)}_{\mathrm{{pdf}}}$:

3 $$\begin{aligned} \chi ^{2(k)}_{\mathrm{{pdf}}}\equiv & {} \sum _{i,j=1}^{N_x} \sum _{\alpha ,\beta =1}^{N_\mathrm{f}}([f_{H,\alpha }^{(k)}(x_i,Q^2_0) - f_{\alpha }^{(k)}(x_i,Q^2_0)]\nonumber \\&\cdot (\mathrm{cov^{pdf}})^{-1}_{ij,\alpha \beta } \cdot [f_{H,\beta }^{(k)}(x_j,Q^2_0) - f_{\beta }^{(k)}(x_j,Q^2_0)]).\nonumber \\ \end{aligned}$$

Note in Eqs. (2) and (3) the use of the subscript “pdf”, to avoid any confusion with the covariance matrix and the $\chi ^2$ in the space of experimental data, which do not play any role here.

The optimal set of expansion coefficients $\{a_i^{(k)}\}$ for each of the original $N_{\mathrm{{rep}}}$ replicas is determined by minimization of Eq. (3). This is a convex problem which can be solved in an efficient way through Singular Value Decomposition (SVD) techniques. The problem consists of finding the vector ${\vec {a}}$ of dimension $N_{\mathrm{{eig}}}$ that minimizes the residual of a linear system of dimensions $(N_{x}N_{f})\times N_{\mathrm{{eig}}}$ for each replica of the original set. The PDF covariance matrix Eq. (2) can be viewed as a $(N_{x}N_{f})\times (N_{x}N_{f})$ matrix $\mathrm{cov}^{\mathrm{{pdf}}}_{lm}$, with indices *l*, *m* related to those of the original definition by $l=N_x(\alpha -1) + i$ and $m=N_x(\beta -1) + j$. Then we define an $N_xN_{\mathrm{{pdf}}}\times N_{\mathrm{{eig}}}$ matrix $Y_{mq}$ as

4 $$\begin{aligned} Y_{mq} = \eta ^{(q)}_\beta (x_j), \end{aligned}$$

with the same definition for the index *m*. We can now lay out the linear system by defining

5 $$\begin{aligned} A_{lq}= & {} \sum _{m=1}^{N_{x}N_{\mathrm{{pdf}}}}(\mathrm{cov}^{\mathrm{{pdf}}})^{-{{1}\over {2}}}_{lm} Y_{mq},\nonumber \\ b_l^{(k)}= & {} \sum _{m=1}^{N_{x}N_{\mathrm{{pdf}}}}(\mathrm{cov}^{\mathrm{{pdf}}})^{-{{1}\over {2}}}_{lm} f_{\alpha }^{(k)}(x_i), \end{aligned}$$

again, with $l=N_x(\alpha -1) + i$. Here $(\mathrm{cov}^{\mathrm{{pdf}}})^{-{{1}\over {2}}}$ stands for a square root of inverse covariance matrix, i.e., for a semi-positive definite real matrix *A*, the matrix such that $(A^{{{1}\over {2}}})^tA^{{{1}\over {2}}}=A$. Finally we can recast the original problem Eq. (3) as that of finding ${\vec {a}}$ that minimizes $\Vert A{\vec {a}}-b^{(k)}\Vert$.

If the starting number of MC replicas is large enough, they will not all be linearly independent. In such case, if the number of eigenvectors $N_{\mathrm{{eig}}}$ is too large, the system will be over-determined and the solution will be degenerate in the space of linear expansion coefficients ${\vec {a}}$. In these conditions, the correlations between this parameters will be ill-defined, and will result in a numerically unstable covariance matrix Eq. (6). On the other hand, if the number of eigenvectors $N_{\mathrm{{eig}}}$ is too small, it will not be possible to achieve a small value of the figure of merit Eq. (3) and the Hessian representation of the original covariance matrix will be a poor approximation. Therefore, on quite general grounds one expects that, if one starts with an extremely large (“infinite”) number of MC replicas, there will always be an optimal value of $N_{\mathrm{{eig}}}$.

In Eq. (3) we have introduced a sampling in *x*, with a total of $N_x$ points. This immediately raises the issue of choosing both a suitable spacing and range of the grid of points in *x*. Because PDFs are generally quite smooth, neighboring points in *x* are highly correlated, and thus the *x*-grid cannot be too fine-grained, otherwise the matrix $\mathrm {cov}^{\mathrm{{pdf}}}$ rapidly becomes ill-conditioned. Furthermore, the choice of the *x*-grid range must keep into account not only the fact that we want the replicas to be especially well-reproduced where they are accurately known (hence the grid should not be dominated by points in extrapolation regions), but also that the whole procedure is meaningful only if the starting probability distribution is at least approximately Gaussian. The way both issues are handled will be discussed in detail in Sect. 2.2 below.

Having determined the expansion coefficients $\{a_i^{(k)}\}$, we obtain the eigenvector directions which describe our original replica set by computing their covariance matrix:

6 $$\begin{aligned} \mathrm{cov}^{(a)}_{ij}= & {} {{N_{\mathrm{{rep}}}}\over {N_{\mathrm{{rep}}}-1}} (\langle a_i^{(k)} a_j^{(k)}\rangle _{\mathrm{{rep}}} - \langle a_i^{(k)}\rangle _{\mathrm{{rep}}} \langle a_j^{(k)}\rangle _{\mathrm{{rep}}} ),\nonumber \\&i,j=1,\ldots ,N_{\mathrm{{eig}}}. \end{aligned}$$

This covariance matrix in the space of the linear expansion parameters Eq. (6) should not be confused with the covariance matrix in the space of PDFs, defined in Eq. (2) (hence the different superscripts). The Hessian matrix is then the inverse of $\mathrm{cov}^{(a)}_{ij}$, which we can diagonalize through a rotation matrix $v_{ij}$, thus obtaining a set of eigenvalues $\lambda _i$ (as in the Meta-PDF method of Ref. [13]).

We thus obtain the one-sigma uncertainty band associated to each orthogonal direction by normalizing by $\sqrt{\lambda _i}$. Therefore the total one-sigma uncertainty will be given by

7 $$\begin{aligned}&\sigma ^\mathrm{PDF}_{H,\alpha }(x,Q^2) \nonumber \\&\quad =\sqrt{\sum _{i=1}^{N_{\mathrm{{eig}}}}\left[ \sum _{j=1}^{N_{\mathrm{{eig}}}} {{v_{ij}}\over {\sqrt{\lambda _i}}}(\eta ^{(j)}_{\alpha }(x,Q^2)-f^{(0)}_{\alpha }(x,Q^2) )\right] ^2}, \end{aligned}$$

and our final Hessian representation of the original Monte Carlo PDF set is composed by $N_{\mathrm{{eig}}}$ symmetric eigenvectors, given by

8 $$\begin{aligned} \widetilde{f}_{\alpha }^{(i)}(x,Q^2)= & {} f^{(0)}_{\alpha }(x,Q^2)+ \sum _{j=1}^{N_{\mathrm{{eig}}}}{{v_{ij}}\over {\sqrt{\lambda _i}}}\nonumber \\&\times (\eta ^{(j)}_{\alpha }(x,Q^2)-f^{(0)}_{\alpha }(x,Q^2)). \end{aligned}$$

For PDF sets obtained with this Hessian representation, one should use the symmetric Hessian formula, namely, the one-sigma PDF uncertainty will be given by

9 $$\begin{aligned} \sigma ^\mathrm{PDF}_{H,\alpha }(x,Q^2) = \sqrt{ \sum _{i=1}^{N_{\mathrm{{eig}}}} (\widetilde{f}_{\alpha }^{(i)}(x,Q^2) - f_{\alpha }^{(0)}(x,Q^2) )^2}, \end{aligned}$$

which is the practical recipe for Eq. (7). An analogous expression should be used for the computation of PDF uncertainties in physical cross-sections.

If the method is successful, Eq. (9) should be close to the original result for the one-sigma PDF uncertainty in the MC representation, namely

10 $$\begin{aligned} \sigma ^\mathrm{PDF}_{\alpha }(x,Q^2) = \sqrt{ \langle ({f}^{(k)}_{\alpha }(x,Q^2))^2\rangle _{\mathrm{{rep}}} - \langle {f}^{(k)}_{\alpha }(x,Q^2)\rangle _{\mathrm{{rep}}}^2 }.\nonumber \\ \end{aligned}$$

Of course, once the Hessian representation is available, all the Hessian technology can be used, like the dataset diagonalization method [9] or the computation of the correlation coefficients between different cross-sections [15]. Likewise, one can now easily include the orthogonal eigenvectors in a nuisance parameter analysis.

### Optimization

We discuss now the determination of the optimal set of parameters which characterize our procedure, and specifically:

- the optimal grid of points in *x* over which the figure of merit Eq. (3) is evaluated;
- the optimal number of eigenvectors $N_{\mathrm{{eig}}}$ and the optimal choice of the basis replicas.

We consider in particular the application of our method to a prior set of $N_{\mathrm{{rep}}} = 1000$ MC replicas from the NNPDF3.0 NLO set. We then consider also $N_{\mathrm{{rep}}} = 1000$ MC replicas of the MMHT14 NLO set, constructed from the original Hessian representation using the Hessian to Monte Carlo conversion methodology of Ref. [10].

A suitable choice for the grid of points in *x* is one that ensures that PDFs are well-reproduced in the kinematic region which is relevant for phenomenology, and that the spacing of the grid is such that correlations between neighboring points are not so strong that it becomes impossible to invert the covariance matrix. In practice, we proceed as follows: we first consider an initial grid of points in $x\in [10^{-5},0.9]$ with $N_{x}=50$ for all PDFs ($N_{\mathrm{{pdf}}}=7$, since heavy flavor PDFs are generated dynamically), half equally spaced on a logarithmic scale for $x\in [10^{-5},10^{-1}]$ and half equally spaced on a linear scale for $x\in [0.1,0.9]$. We then determine the eigenvectors of the ensuing $350\times 350$ ($N_x N_{\mathrm{{pdf}}}\times N_x N_{\mathrm{{pdf}}}$) covariance matrix, and discard all eigenvectors corresponding to eigenvalues whose size is smaller than a factor $10^{-12}$ times the largest one. This removes points which carry little information due to large correlations. We then invert the covariance matrix in the remaining subspace.

**Fig. 1:**
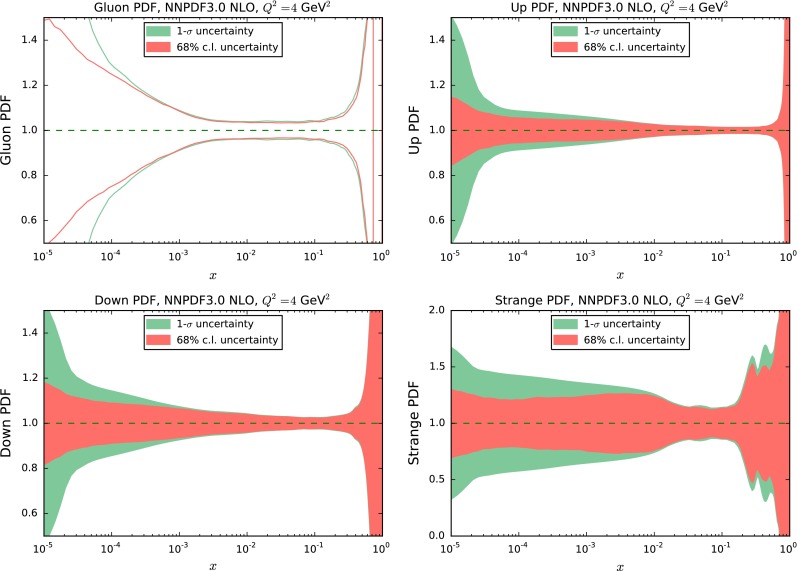
Comparison of one-sigma and 68 % confidence level intervals for some PDFs from the NNPDF3.0 NLO set, determined using a sample of $N_{\mathrm{{rep}}}=1000$ MC replicas, at $Q=4$ GeV$^2$. From *top to bottom* and from *left to right* the gluon, down, up and strange PDFs are shown

A further difficulty arises whenever the prior uncertainties are not Gaussian. In such case, a faithful Hessian representation is (by construction) impossible, and our procedure, which always leads to a final Hessian matrix, becomes meaningless. Whenever the starting PDF set has potentially non-Gaussian uncertainties, it is thus necessary to quantify the deviation from gaussianity in order to make sure that the procedure can be consistently applied. We do this using the simplest indicator, namely the second moment of the probability distribution, specifically comparing the one-sigma and 68 % confidence level intervals [16], which for a Gaussian distribution coincide. The comparison is shown in Fig. 1 for some PDFs in the NNPDF3.0 NLO set at $Q^2 = 4$ GeV$^2$. It is clear that deviations from gaussianity can be significant whenever experimental information is scarce or missing, specifically at small- and large-*x*, since in these regions the PDF uncertainty is not determined by gaussianly distributed data but rather by extrapolation and by theoretical constraints (such as sum rules and cross-section positivity).

We thus define the indicator

11 $$\begin{aligned} \epsilon _{\alpha }(x_i,Q^2_0) = {{\left| \sigma _{\alpha }(x_i,Q^2_0) - \sigma ^{68}_{\alpha }(x_i,Q^2_0)\right| }\over {\sigma ^{68}_{\alpha }(x_i,Q^2_0)}}, \end{aligned}$$

where $\sigma _{\alpha }(x_i,Q^2_0)$ and $\sigma _{\alpha }^{68}(x_i,Q^2_0)$ are respectively the one-sigma and 68 % confidence level intervals for the $\alpha$-th PDF at point $x_i$ and scale $Q^2_0$, computed from the original MC representation with $N_{\mathrm{{rep}}}=1000$ MC replicas. When the prior set has potentially non-Gaussian uncertainties, first of all we evaluate the figure of merit Eq. (11) on the same grid of point on which the covariance matrix is computed. We then discard all points for which the deviation Eq. (11) exceeds some threshold value $\epsilon$, i.e. we only include points such that

12 $$\begin{aligned} \epsilon _{\alpha }(x_i,Q^2_0)<\epsilon . \end{aligned}$$

We then proceed as above: on the remaining points we compute the covariance matrix, determine its eigenvectors, and discard eigenvectors whose size is less than twelve orders of magnitude smaller than the largest eigenvector. Needless to day, this additional initial step is not required for sets (such as MMHT14) which are obtained from a Monte Carlo conversion of an original Hessian set, and thus have Gaussian uncertainties by construction.

We now turn to the determination of the optimal basis of replicas for the Hessian representation. This optimization requires the definition of a statistical estimator which measures the quality of the Hessian representation. Given that the Hessian representation corresponds to a Gaussian distribution, and that central values are reproduced by construction, the probability distribution is fully determined by the covariance matrix. In practice, however, a good assessment of the quality of the Hessian representation is obtained by simply verifying that the diagonal elements of the covariance matrix are well reproduced, thanks to the fact that correct correlations are automatically provided by the use of PDF replicas as a basis, as we shall explicitly verify below.

We introduce therefore the estimator

13 $$\begin{aligned} {\text {ERF}}_{\sigma } = \sum _{i=1}^{N_x} \sum _{\alpha =1}^{N_{\mathrm{{pdf}}}} \left| {{\sigma ^\mathrm{{PDF}}_{H,\alpha }(x_i,Q^2_0) - \sigma ^\mathrm{{PDF}}_{\alpha }(x_i,Q^2_0)}\over {\sigma ^\mathrm{{PDF}}_{\alpha }(x_i,Q^2_0)}}\right| , \end{aligned}$$

which compares the one-sigma standard deviations computed for the original MC and their Hessian representations, as given respectively by Eqs. (10) and (9). We then compute the estimator for a given fixed value of $N_{\mathrm{{eig}}}$ basis replicas. The choice of these is random at first, and then optimized using a Genetic Algorithm (GA).

The parameters of the GA are chosen based on the studies of Ref. [14], where the related problem of optimizing the choice of PDF replica set was studied: we find that a single mutation per iteration of the GA is sufficient, with the number of mutants chosen to be between one and four per mutation, with probabilities listed in Table 1. It turns out that $N_{\mathrm{{gen}}}^{\mathrm{{max}}}=2000$ iterations of the GA are sufficient to obtain good stability and a sizable improvement of the figure of merit in comparison to the starting random selection.

**Table 1:** Number of mutants per replica and respective probabilities for each generation of the GA

mc2hessian v1.0.0	
$N_{\mathrm{{rep}}}^{\mathrm{{mut}}}$	$P_{\mathrm{{mut}}}$ (%)
1	30
2	30
3	10
4	30

**Fig. 2:**
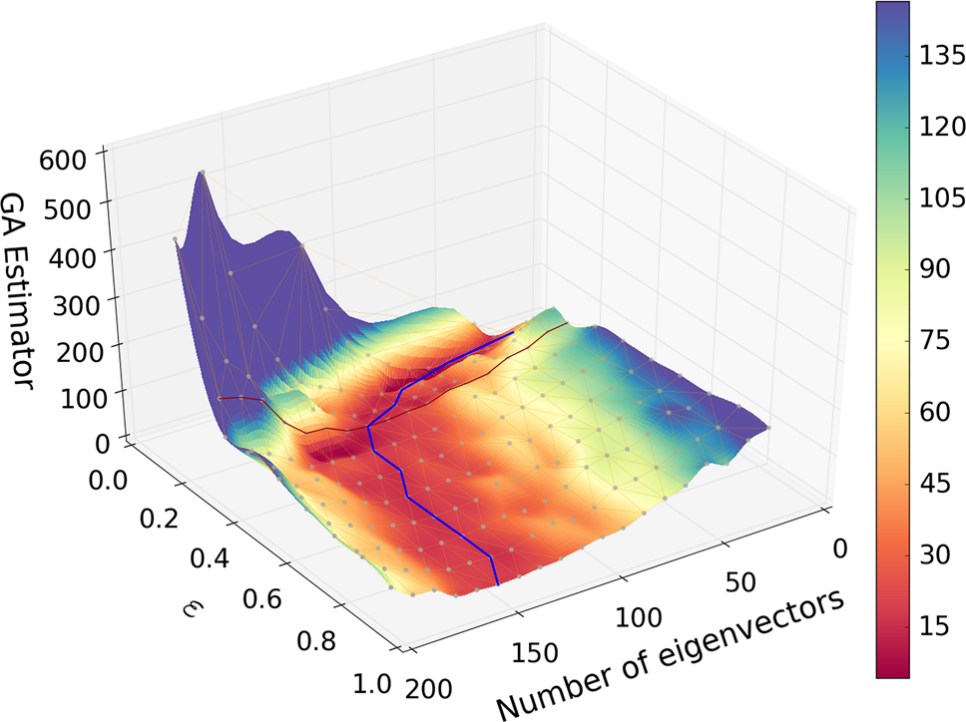
The figure of merit, Eq. (13), for the Hessian representation of the NNPDF3.0 NLO set, computed for points which satisfy the gaussianity criterion Eq. (12), plotted versus the threshold $\epsilon$ Eq. (12) and the number of eigenvectors $N_{\mathrm{{eig}}}$, after the choice of basis replicas has been optimized through a run of the GA with the settings of Table 1. The value of the estimator along the valley of minima, i.e. the curve determined by finding the value of $N_{\mathrm{{eig}}}$ at which the estimator has its absolute minimum for each $\epsilon$ is shown (*blue curve*). *The red curve* marks the value $\epsilon =0.25$ which is finally adopted. The projection of the valley of minima in the $(\epsilon , N_{\mathrm{{eig}}})$ plane is shown in Fig. 3

In Fig. 2 we plot the value of the estimator Eq. (13) after GA minimization, as a function of the threshold value of $\epsilon$ and the number $N_{\mathrm{{eig}}}$ of basis replicas. A valley of minima is clearly seen, and shown in the plot as a curve, determined by searching for the absolute minimum of the estimator as a function of $N_{\mathrm{{eig}}}$ for each fixed $\epsilon$.

**Fig. 3:**
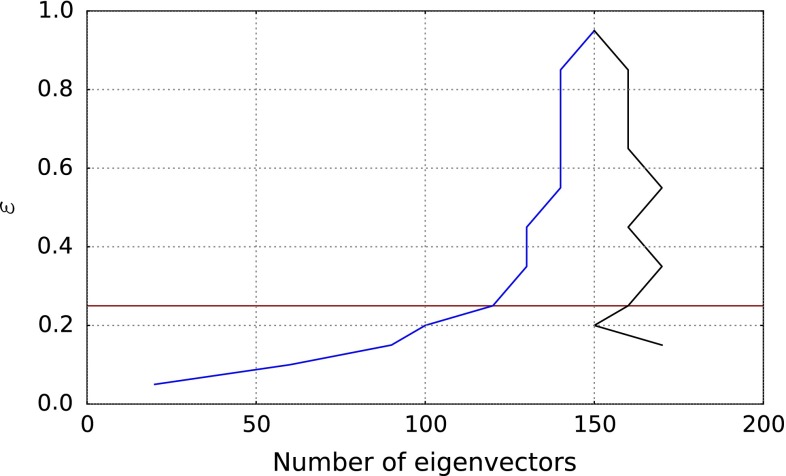
The valley of minima shown in Fig. 2 (*blue curve*) shown as a projection on the $(\epsilon , N_{\mathrm{{eig}}})$ plane, compared to the curve recomputed using the same eigenvector basis but including all points in the determination of the figure of merit (*black curve*). *The red line* indicates the threshold value $\epsilon =0.25$ which is finally adopted

An interesting feature of Fig. 2 is that for all values of $\epsilon$ the optimal value of $N_{\mathrm{{eig}}}$ is reasonably small, and much smaller than the total number of replicas $N_{\mathrm{{rep}}}=1000$. As one might expect, when the value of the threshold $\epsilon$ is small, and thus a large number of points in *x* is excluded, the optimal number of eigenvectors is also small, rapidly decreasing when $\epsilon \lesssim 0.2$. Clearly, however, if $\epsilon$ is too small, only few points will be retained in the computation of the figure of merit Eq. (3) and the original MC replicas will not be reproduced accurately enough.

In order to determine the optimal value of $\epsilon$, for each value of $N_{\mathrm{{eig}}}$ and $\epsilon$ shown in Fig. 2 we have recomputed the figure of merit Eq. (13) using the same eigenvector basis, but now including all points; the valley of minima is then determined again for this new surface. The dependence on $\epsilon$ is now due to the fact that the eigenvector basis changes as $\epsilon$ is varied, though the definition of the figure of merit does not. Therefore, the difference between the two curves is a measure of how much the exclusion of nongaussian points by the $\epsilon$ criterion affects the choice of optimal eigenvector set. The two curves are compared in Fig. 3: they are seen to diverge when $\epsilon \lesssim 0.2$. We take this as an indication that, below this value, the amount of information which is necessary in order to describe all points starts being significantly different from that which is sufficient for an accurate description of the points which have passed the cut, and thus the cut becomes too restrictive. We consequently adopt $\epsilon =0.25$ (red curve), as a reasonable compromise between only including points for which uncertainties are Gaussian, and not loosing too much information.

**Fig. 4:**
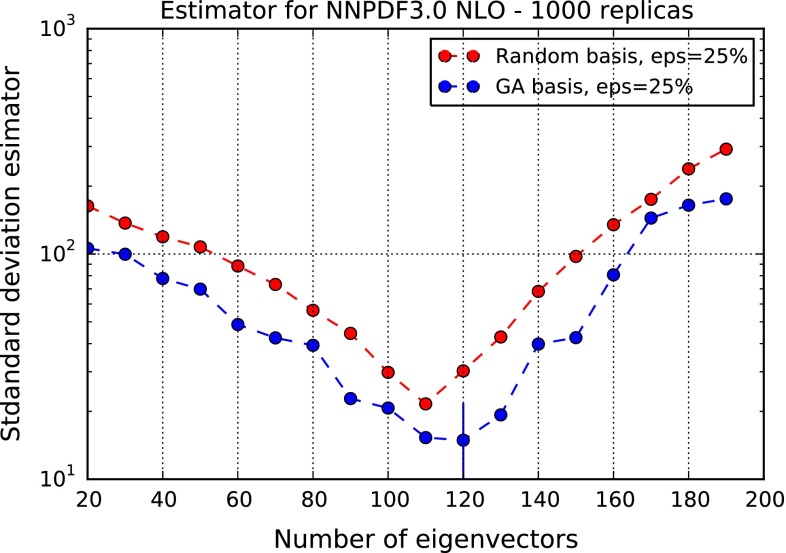
The figure of merit of Fig. 2, plotted versus $N_{\mathrm{{eig}}}$ for fixed $\epsilon =0.25$ (*blue curve*), for the NNPDF3.0 NLO set. The optimal value $N_{\mathrm{{eig}}}=120$ is denoted by *a vertical dash*. Results obtained by not optimizing the basis through the GA are also shown (*red curve*)

The profile of the figure of merit with the choice of threshold value $\epsilon =0.25$, is shown in Fig. 4. It is seen that the optimal number of eigenvectors is $N_{\mathrm{{eig}}} = 120$. The fact that this value is much less than the starting $N_{\mathrm{{rep}}}=1000$ means that replicas in the original set are strongly correlated. This is nicely consistent with the result that it is possible to construct a “compressed” representation of NNPDF3.0, in which the original probability distribution is reproduced but including a much smaller, optimized set of Monte Carlo replicas [14]. In fact, it turns out that the optimal number of eigenvectors, and the number of compressed replicas, are of the same order of magnitude.

In Fig. 4 we also show the figure of merit when the basis replicas are chosen randomly, instead of being optimized through the GA. It is apparent that use of the GA leads to an improvement of the figure of merit by almost a factor two. It is interesting to observe that this improvement is in fact achieved by modifying only a small fraction of the initial random selection of replicas: specifically, only 26 of the replicas used as initial input for the basis are mutated at the end of the $N_{\mathrm{{gen}}}^{\mathrm{{max}}}$ iterations of the GA. This suggests that there is still room for improvement in the selection of the optimal basis, since the GA only explores combinations that are not to far from the initial basis.

**Fig. 5:**
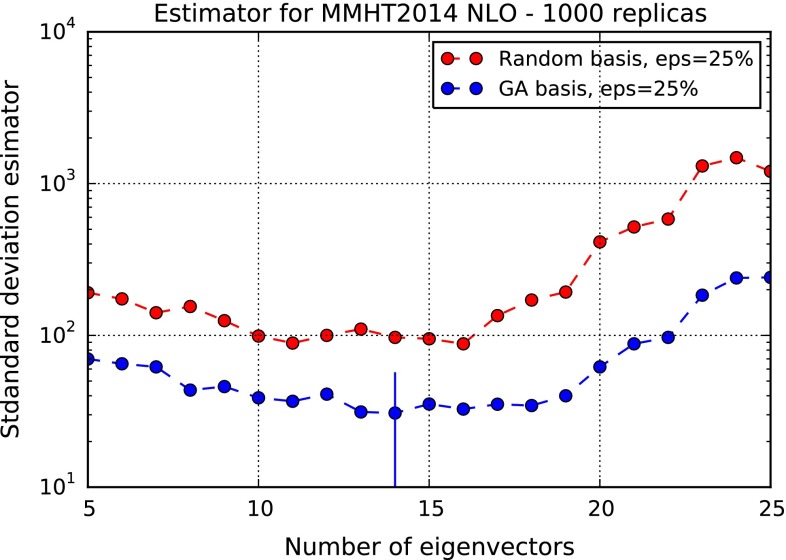
Same as Fig. 4, but for the MMHT14 MC NLO PDF set. In this case the gaussianity condition Eq. (12) is not applied. The optimal value $N_{\mathrm{{eig}}}=14$ is denoted by a *vertical dash*

Finally, we have repeated our construction for the Monte Carlo representation of the MMHT14 NLO PDF set. In this case, because the starting PDF set is Hessian, no gaussianity requirements are necessary. On the other hand, because the underlying PDF set is described by a smaller number of parameters than either the NNPDF or the combined set considered previously, Monte Carlo replicas tend to be more correlated. As a consequence, it is necessary to relax somewhat the criterion for selection of the eigenvectors of the covariance matrix: in this case, we keep all eigenvectors corresponding to eigenvalues whose size is larger than $10^{-15}$ times that of the largest one (instead of $10^{-12}$ as in the previous cases). We then simply determine the figure of merit as a function of $N_{\mathrm{{eig}}}$: results are shown in Fig. 5. Also in this case, the GA leads to an improvement of the figure of merit by more than a factor two. Now, however, the optimal number of eigenvectors is rather smaller, $N_{\mathrm{{eig}}} = 14$, as compared to the NNPDF3.0 result. Again, this is of the same order as that which is used when applying the compression algorithm of Ref. [14] to the MMHT14 PDFs.

## Results and validation

We now study in detail the results which are obtained when applying our Monte Carlo to Hessian conversion to NNPDF3.0 NLO – a native Monte Carlo PDF set – and MMHT14 – a Hessian PDF set which has been turned into Monte Carlo using the technique of Ref. [10]. In the former case, we compare the final Hessian PDFs to the starting Monte Carlo ones, at the level of central values, uncertainties, and correlations, thereby validating the procedure. In the latter case, we compare the results of the final Hessian conversion to the original Hessian set, thereby providing a powerful closure test of the procedure. Finally, we compare results before and after Hessian conversion for both PDF sets at the level of physical observables, in order to ascertain the accuracy of our methodology for realistic applications. PDF comparisons and luminosity plots shown in this section have been produced using the APFEL Web plotting tool [17, 18].

### Hessian representation of Monte Carlo PDFs

**Fig. 6:**
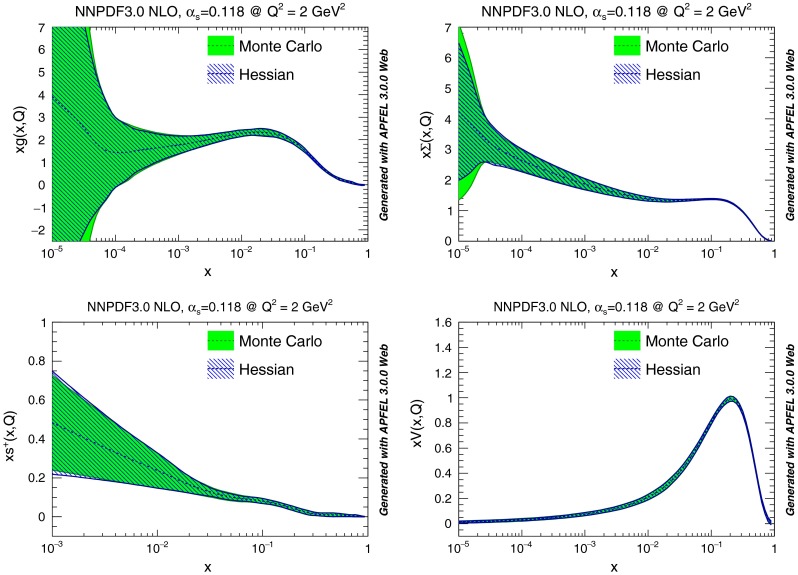
Comparison between the starting Monte Carlo representation with $N_{\mathrm{{rep}}}=1000$ replicas and the final Hessian representation with $N_{\mathrm{{eig}}}=120$ eigenvectors for the for the NNPDF3.0 NLO set with $\alpha _s=0.118$. From *left to right* and from *top to bottom* we the gluon, total quark singlet, total strangeness and the total valence are plotted vs. *x* for fixed $Q^2=2$ GeV$^2$

**Fig. 7:**
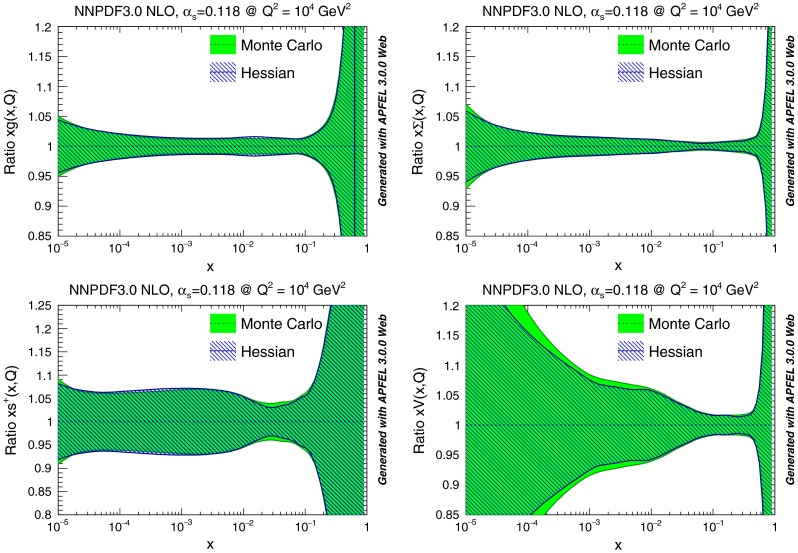
Same as Fig. 6 but at $Q^2=10^4$ GeV$^2$, and with results normalized to the central PDF

We concentrate on the PDF set obtained starting with $N_{\mathrm{{rep}}}=1000$ NNPDF3.0 NLO replicas, and applying our methodology with the optimal choice of parameters discussed in Sect. 2.2, namely $\epsilon =0.25$, $N_{\mathrm{{eig}}} = 120$. In Fig. 6 we compare the original Monte Carlo representation to the final Hessian representation of several PDFs at $Q^2=2$ GeV$^2$: the excellent accuracy of the Hessian representation is apparent, with differences in the one-sigma PDF uncertainty bands of the order 5 % at most (recall that central values coincide by construction). In Fig. 7 the same comparison is performed at $Q^2=10^4$ GeV$^2$, now shown as a ratio to central values.

**Fig. 8:**
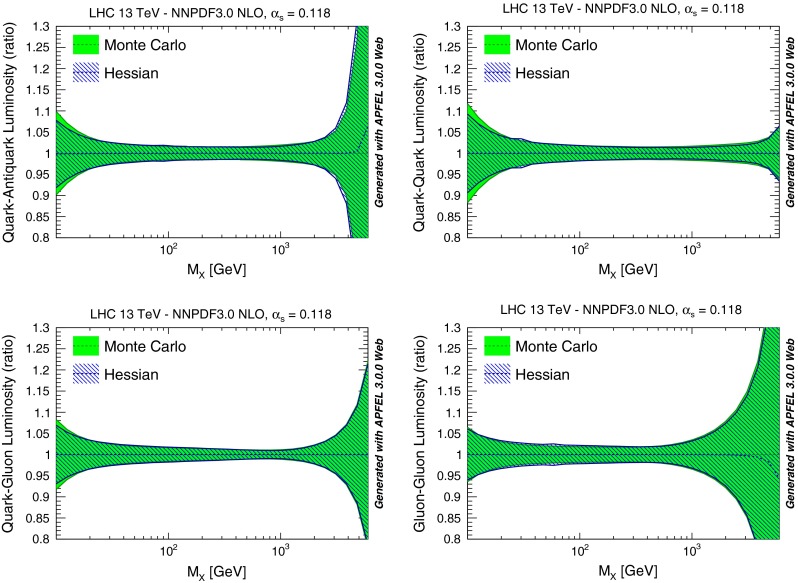
Same as Fig. 6, but now comparing parton luminosities for proton–proton collisions at 13 TeV, plotted vs. the invariant mass of the final state. Results are shown normalized to the central PDF

In Fig. 8 we then compare some parton luminosities, computed for proton–proton collisions with a center of mass energy of 13 TeV, plotted vs. the invariant mass of the final state. Results are shown normalized to the central value of the NNPDF3.0 NLO Monte Carlo set. Again, we find excellent agreement, except in the regions of very small or very large invariant masses (which respectively depend on small and large-*x* PDFs). This is unsurprising given that these are extrapolation regions in which the Gaussian approximation is less good.

As mentioned in Sect. 2.2, even though the figure of merit Eq. (13) used for the GA only optimizes the diagonal elements of the covariance matrix, correlations are automatically reproduced thanks to the use of the original replicas as a basis. We can check this explicitly. The correlation coefficient between two different PDFs $f_{\alpha }$ and $f_\beta$, at a given value of *x*, $Q^2$, in the Monte Carlo representation is given by [19]:

14 $$\begin{aligned}&\rho ^{\alpha \beta }_\mathrm{MC}(x,Q^2) = {{N_{\mathrm{{rep}}}}\over {N_{\mathrm{{rep}}}-1}}\nonumber \\&\quad \times \left( {{\langle f_{\alpha }^{(k)}(x,Q^2)f_\beta ^{(k)}(x,Q^2)\rangle _{\mathrm{{rep}}}- \langle f_{\alpha }^{(k)}(x,Q^2)\rangle _{\mathrm{{rep}}}\langle f_\beta ^{(k)}(x,Q^2)\rangle _{\mathrm{{rep}}}}\over {\sigma ^\mathrm{PDF}_{\alpha }(x,Q^2) \cdot \sigma ^\mathrm{PDF}_\beta (x,Q^2) }}\right) ,\nonumber \\ \end{aligned}$$

where averages are taken over the $N_{\mathrm{{rep}}}=1000$ replicas of the sample, and $\sigma _{\alpha }(x,Q^2)$ and $\sigma _\beta (x,Q^2)$ are the standard deviations Eq. (10). In the Hessian representation the same quantity is given by

15 $$\begin{aligned} \rho ^{\alpha \beta }_{\mathrm{{Hessian}}}(x,Q^2) ={{\sum _{k=1}^{N_{\mathrm{{eig}}}}[(\widetilde{f}_{\alpha }^{(k)}(x,Q^2) - f_{\alpha }^{(0)}(x,Q^2))(\widetilde{f}_\beta ^{(k)}(x,Q^2) - f_\beta ^{(0)}(x,Q^2))]}\over {\sqrt{\sum _{k=1}^{N_{\mathrm{{eig}}}}(\widetilde{f}_{\alpha }^{(k)}(x,Q^2) - f_{\alpha }^{(0)}(x,Q^2) )^2} \sqrt{\sum _{k=1}^{N_{\mathrm{{eig}}}}(\widetilde{f}_\beta ^{(k)}(x,Q^2) - f_\beta ^{(0)}(x,Q^2) )^2}}}, \end{aligned}$$

where now the sum is performed over the $N_{\mathrm{{eig}}}$ eigenvectors, and $f_{\alpha }^{(0)},f_\beta ^{(0)}$ are the respective central sets, which coincide with the MC average values.

**Fig. 9:**
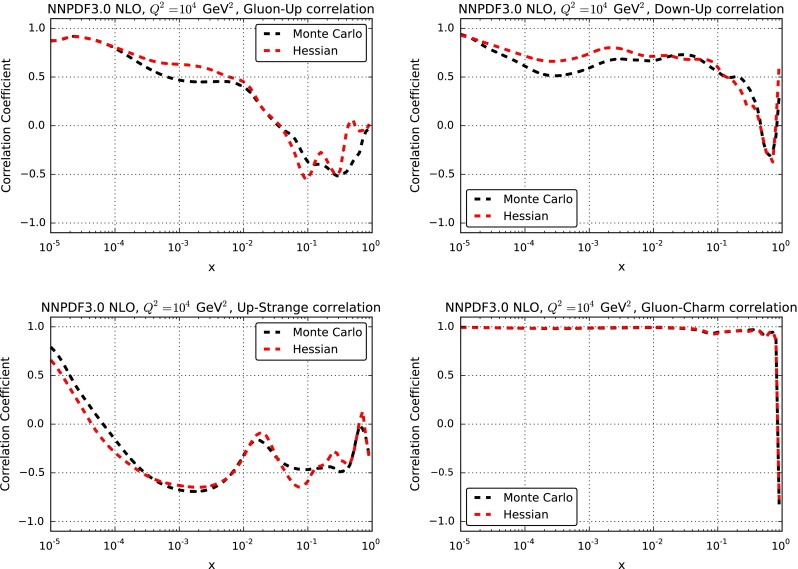
Correlation coefficients between pairs of PDFs at a common value of *x* and $Q^2$ versus *x* for $Q^2=10^4$ GeV$^2$ before and after Hessian conversion

The correlation coefficients Eqs. (14) and (15) before and after Hessian conversion are compared in Fig. 9: again, very good agreement is seen, with differences compatible with the uncertainty on the Monte Carlo representation. We have checked explicitly that a similar level of agreement is found at the level of correlations between a number of LHC cross-sections and differential distributions.

We conclude that at the level of second moments the Hessian representation of the starting Monte Carlo probability distribution is very accurate. Of course, to the extent that higher moments deviate from Gaussian behaviour they will be accordingly not so well reproduced.

### A closure test: Hessian representation of Hessian PDFs

We now consider the MMHT14 NLO PDF set. In this case, the Monte Carlo representation which is converted into Hessian is in turn obtained by starting from an initial Hessian representation, using the methodology of Ref. [10]. We can then compare the starting and final Hessian sets, thereby obtaining a closure test. This provides a powerful test of the basis-independence of our procedure, in that the starting Hessian is defined in the space of parameters of a specific functional form, which is then turned into Hessian by running a Monte Carlo in parameter space, while our final Hessian representation uses the ensuing Monte Carlo replicas as basis functions.

Again, we adopt the optimal choice of parameters discussed in Sect. 2.2, namely $N_{\mathrm{{eig}}} = 14$. Note that this was obtained by relaxing somewhat the criterion for keeping eigenvectors of the covariance matrix, due to the greater correlation of MMHT PDF replicas: indeed, we have verified that use of the same criterion as for the sets we considered previously would need to a smaller optimal number of eigenvectors ($N_{\mathrm{{eig}}} = 12$ instead of $N_{\mathrm{{eig}}} = 14$), and a considerable loss of accuracy.

**Fig. 10:**
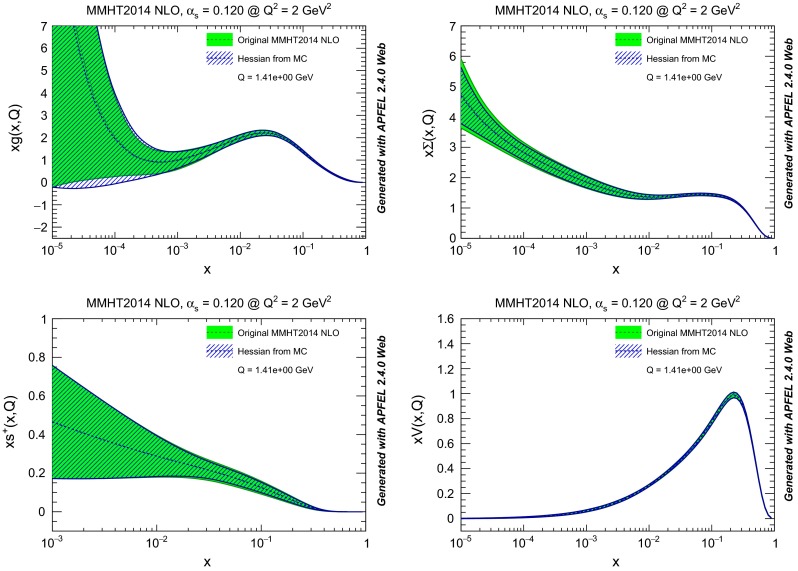
Same as Fig. 6, but for MMHT14 NLO PDFs, with $N_{\mathrm{{eig}}}=25$ asymmetric eigenvectors in the starting Hessian set and $N_{\mathrm{{eig}}}=14$ symmetric eigenvectors in our final Hessian conversion

The starting Hessian representation and our final Hessian conversion are compared in Fig. 10 at $Q^2=2$ GeV$^2$. Again we find agreements of the uncertainty to better than 5 %. It is interesting to observe that the original Hessian representation had $N_{\mathrm{{eig}}}=25$ asymmetric eigenvectors (corresponding to 50 error sets), while our final Hessian conversion only needs $N_{\mathrm{{eig}}}=14$ symmetric eigenvectors. This means that our algorithm has managed to achieve a compression of the information in the native Hessian representation, thanks to the use of replicas as a basis, with minimal information loss.

### LHC phenomenology

We finally validate our Hessian conversion at the level of physical observables: standard candle total cross-sections and differential distributions. For simplicity, we perform all comparisons at NLO, given that, clearly, the accuracy of the Hessian approximation is essentially independent of the perturbative order.

**Fig. 11:**
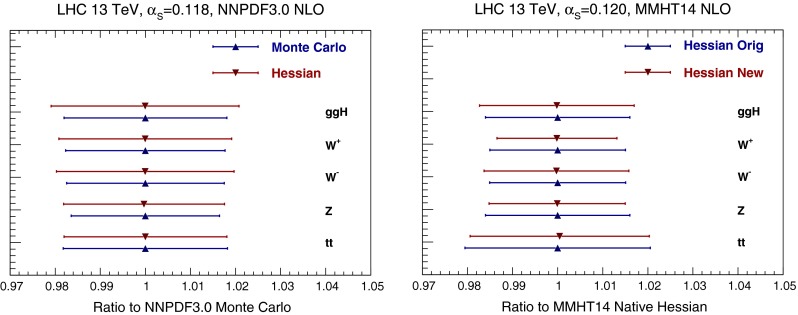
Comparison of NLO inclusive cross-sections at the LHC, computed using PDFs before and after Hessian conversion: for NNPDF3.0 (*left*) the Hessian representation is compared to the original Monte Carlo, while for MMHT14 (*right*) the final Hessian conversion is compared to the original Hessian

In Fig. 11 we compare results obtained using the Monte Carlo and Hessian NNPDF3.0 representations, and the original and final Hessian representation of MMHT14 PDFs, for the total cross-sections for Higgs production in gluon fusion obtained using the ggHiggs code [20], top quark pair production obtained with top++ [21], and inclusive *W* and *Z* production obtained with VRAP [22]. Results are always shown normalized to the value of the original Monte Carlo set. For NNPDF3.0, the agreement is very good, with PDF uncertainties consistent with 10 % differences at most. Somewhat larger differences are found for MMHT14.

**Fig. 12:**
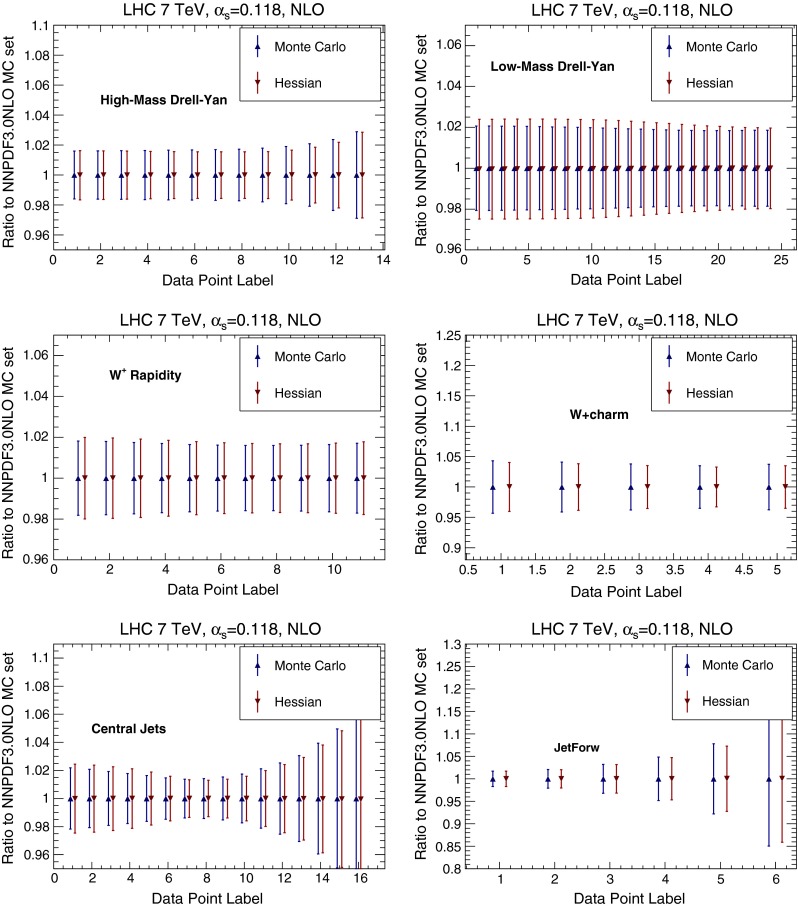
Comparison of the original Monte Carlo representation and the new Hessian representation of NNPDF3.0 NLO for a number of differential distributions at the LHC 7 TeV. The *error band* corresponds to the one-sigma PDF uncertainty in each bin. Results are shown normalized to the central value of the NNPDF3.0 NLO Monte Carlo set. See text for more details

We then compare several differential distributions, chosen among those which have been used in the NNPDF3.0 PDF determination, and for which fast interfaces are available, either APPLgrid [23], FastNLO [24] or aMCfast [25]. Again, results are always shown normalized to the central value of either NNPDF3.0 NLO Monte Carlo set or the MMHT14 NLO native Hessian. In particular we consider:

- The ATLAS high-mass Drell–Yan measurement [26], integrated over rapidity $|y_{ll}|\le 2.1$, and binned as a function of the di-lepton invariant mass pair $M_{ll}$,
- the CMS double differential Drell–Yan measurement [27] in the low-mass region, $20 \ge M_{ll} \ge 30$ GeV, as a function of the di-lepton rapidity $y_{ll}$,
- The CMS $W^+$ lepton rapidity distribution [28],
- The CMS measurement of $W^+$ production in association with charm quarks measurement [29], as a function of the lepton rapidity $y_l$,
- The ATLAS inclusive jet production measurement [30] in the central rapidity region, $|y_\mathrm{jet}|\le 0.3$, as a function of the jet $p_T$, and
- The same ATLAS inclusive jet production measurement [30] now in the forward rapidity region, $4.0 \le |y_\mathrm{jet}|\le 4.4$, as a function of the jet $p_T$.

These observables probe a wide range of PDF combinations, from light quarks and anti-quarks (Drell–Yan) and strangeness ($W+c$) to the gluon (jets) in a wide range of Bjorken-*x* and momentum transfers $Q^2$.

**Fig. 13:**
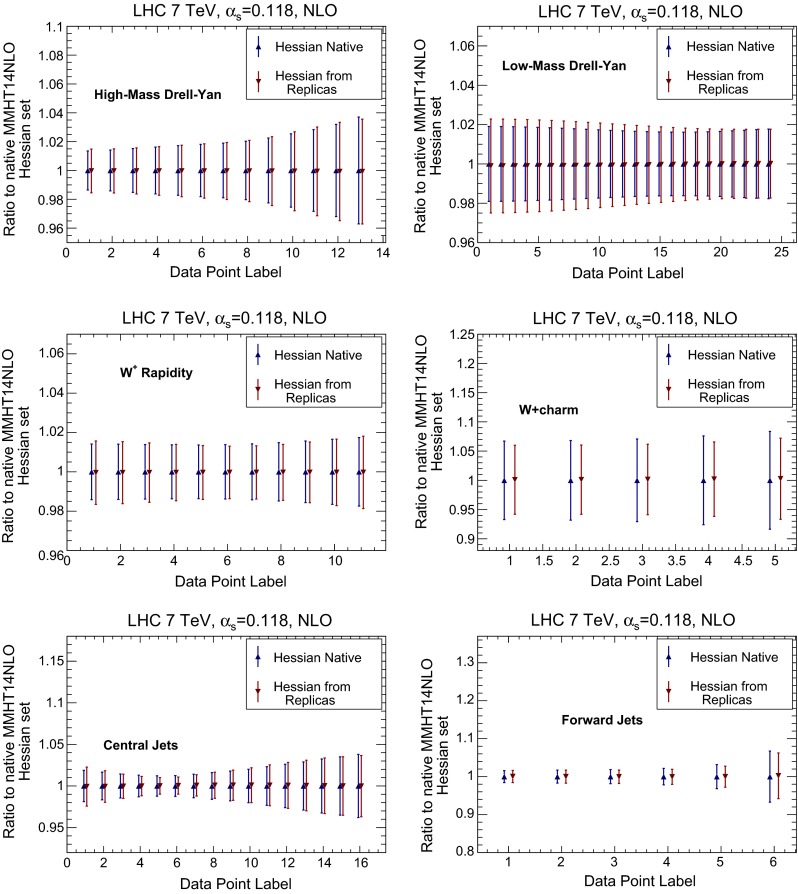
Same as Fig. 12, but for MMHT14 NLO PDFs. The final Hessian set obtained after conversion of the Monte Carlo replicas is compared to the starting Hessian representation

Results are shown in Fig. 12 for NNPDF, and in Fig. 13 for MMHT14. Again, there is a good agreement between the original Monte Carlo and the new Hessian representations, with differences smaller than 10 %.

## A Hessian representation of combined MC sets: MC-H PDFs

As discussed in the introduction, the Monte Carlo representation of PDFs offers the possibility of constructing combined PDF sets which incorporate information from different PDF determinations, and thus provide an alternative [1, 3, 10, 13] to the current PDF4LHC recommendation [11] for the combination of predictions obtained using different PDF sets, which is less than ideal from a statistical point of view.

An obvious shortcoming of a combined Monte Carlo set is that it contains generally a large number of replicas, which can be cumbersome to handle, and which is computationally very intensive. This difficulty has been handled in Ref. [14] by developing a compression algorithm, whereby the number of replicas in a Monte Carlo set is optimized by means of a GA without significant loss of information. This has enabled the construction of sets of less than $N_{\mathrm{{rep}}}=50$ replicas which reproduce most of the information contained in a starting $N_{\mathrm{{rep}}}=300$ replica set.

Combined Monte Carlo set are generally non-Gaussian even when obtained by combining individually Gaussian PDF sets. However, once again the Gaussian approximation may often be adequate in practice, and then a Hessian representation may be useful for applications as repeatedly mentioned. The so-called Meta-PDF method [13] has been proposed as a way of dealing with this problem: it consists of re-fitting a fixed functional form to the final combined Monte Carlo set, and thus it has the usual shortcomings related to a fixed choice of functional form. We now show how by applying our Monte Carlo to Hessian conversion to a combined Monte Carlo set we directly obtained a Hessian representation with a small number of eigenvector, therefore obtaining a compressed Hessian representation, which we call MC-H PDFs.

We start with a Monte Carlo combination of the NNPDF3.0, CT14 and MMHT14 NNLO PDF sets, with $\alpha _s(M_Z)=0.118$. This is the starting point of the construction of the compressed sets of Ref. [14], where further details are given, and it contains $N_{\mathrm{{rep}}} = 300$ replicas.^1^ We could in principle then first, run the compression algorithm of Ref. [14], and then perform a Monte Carlo to Hessian conversion of the ensuing compressed set of replicas. However, each of these two steps entails potential information loss, and thus it is more advantageous to perform directly a Hessian conversion of the starting set of $N_{\mathrm{{rep}}} = 300$ MC replicas.

We thus apply our Monte Carlo to Hessian conversion to the combined prior with $N_{\mathrm{{rep}}}=300$, following the methodology presented in Sect. 2. It actually turns out that significant deviations from Gaussian behavior are observed for PDFs for which direct experimental information is scarce, and thus theoretical bias or constraints play some role, such as the strange PDF. Once a final combined set is made available for phenomenology, a choice will have to be made in order to decide whether a Hessian approximation is viable. For the time being, given the preliminary nature of the existing set, we simply choose $\epsilon =0.25$ as in Sect. 2.2 as a threshold for discarding non-Gaussian points. We then end up with an optimal conversion with $N_{\mathrm{{eig}}} = 90$ eigenvectors, somewhat larger though of the same order than the number of compressed replicas of the CMC set ($N_{\mathrm{{rep}}}\sim 40$).

**Fig. 14:**
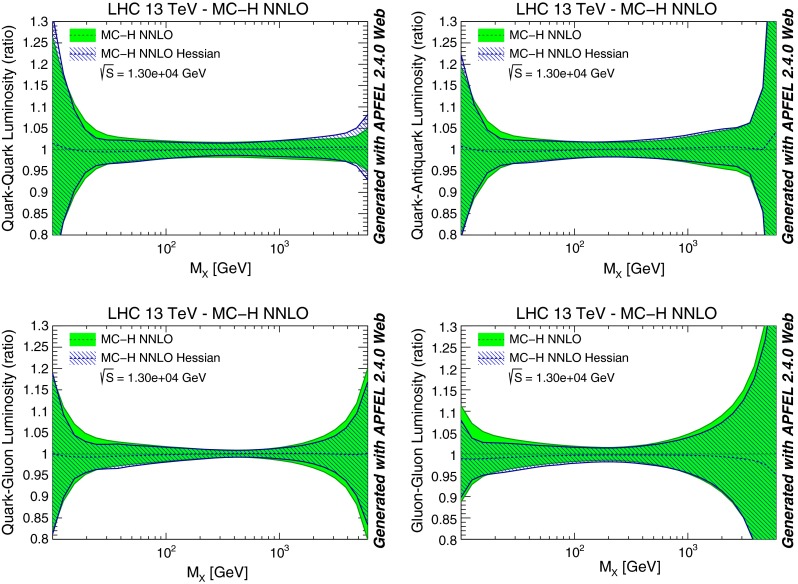
Same as Fig. 8, but now comparing a combined MC PDF set with $N_{\mathrm{{rep}}}=300$ replicas to its MC-H Hessian representation with $N_{\mathrm{{eig}}}=90$ eigenvector. The starting set is obtained from the combination of Monte Carlo representations of the NNPDF3.0, CT14 and MMHT14 NNLO PDF sets containing $N_{\mathrm{{rep}}}=100$ replicas each

We have performed an extensive set of validation tests of these MC-H PDFs, at the level of PDFs, luminosities and physical observables, of which we now show some examples. In Fig. 14 we compare PDF luminosities at the LHC with $\sqrt{s}=13$ TeV computed with the starting combined MC set and the final MC-H Hessian set. We find good agreement, with differences below 10 % at the level of PDF uncertainties. It is important to note that some disagreement is to be expected because the starting combined set is not Gaussian, in particular for regions of *x* (such as large and small *x*) and PDFs that are poorly constrained by experimental data: indeed, the largest discrepancies are observed at low and high invariant masses $M_X$. These differences thus signal an intrinsic limitation of the Hessian representation, rather than a failure of our methodology.

**Fig. 15:**
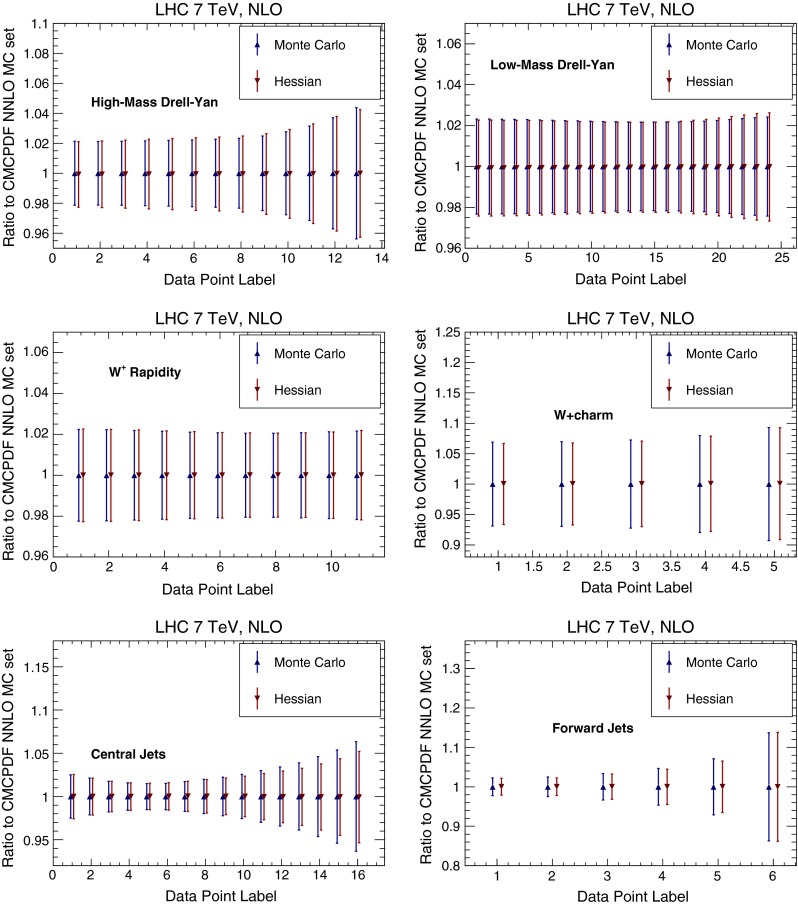
Same as Fig. 12, but now for the PDF sets of Fig. 14

The good agreement at the level of PDF luminosities translates into a good agreement at the level of physical observables. In Fig. 15 we compare the processes that we discussed in Sect. 3.3 (with the same settings), computed using the starting combined MC PDF set and the final MC-H Hessian representation. Again, results are normalized to the central value of the starting combined set, and uncertainties bands correspond to the one-sigma PDF uncertainty in each bin (recall that central values of the starting and final PDF sets are the same by construction). Again, discrepancies are below the 10 % level.

We concluded that the Hessian conversion algorithm presented in this paper also provides a successful methodology for the construction of a Hessian representation with a moderate number of eigenvectors of combined Monte Carlo PDF sets. Differences between the starting MC set and the final MC-H Hessian representation can be used, to a certain extent, to quantify the degree of non-gaussianity which is present in the original set.

## Delivery and outlook

We have provided a general purpose methodology for the Hessian conversion of any Monte Carlo PDF set. When applied to a native Monte Carlo set, this methodology provides an efficient Hessian representation which is faithful to the extent that the starting set is Gaussian. When applied to a Monte Carlo set obtained from a starting Hessian, the methodology gives back the original set to very good accuracy, but using the Monte Carlo replicas as a basis. Finally, when applied to a combined Monte Carlo replica set it provides a Hessian version (MC-H PDFs) of the recently proposed PDF compression methodology (MC-PDFs [14]), and an implementation of the Meta-PDF idea [13] which is free of the bias related to the choice of a specific functional form.

The main deliverable of this work is the mc2hessian code, which easily allows for the construction of a Hessian representation of any given Monte Carlo PDF set. The mc2hessian code is written in Python using the numerical implementations provided by the NumPy/Scipy packages [31]. This code is publicly available from the GitHub repository

https://github.com/scarrazza/mc2hessian

and outputs results directly in the LHAPDF6 format [32], so that the new Hessian sets can be easily interfaced by any other code. However, it should be kept in mind that the Hessian representation always requires careful validation, as some information loss is necessarily involved in this transformation, and specifically any deviation from Gaussianity is inevitably washed out.

The Hessian version of the NNPDF3.0 sets

NNPDF30_nlo_as_0118_hessian

NNPDF30_nnlo_as_0118_hessian

as well as the MC-H PDFs

MCH_nlo_as_0118_hessian

MCH_nnlo_as_0118_hessian

will be made available in LHAPDF6. Future NNPDF releases will be provided both in the native Monte Carlo and in the new Hessian representations.

An interesting development of the methodology suggested here is that an unbiased Hessian representation could be used as a way to single out the PDF flavours (and *x*-ranges) that provide the dominant contribution to individual physics process, by picking the dominant eigenvectors; along the lines of previous suggestions [9, 33], but in a parametrization-independent way. This could then be used to construct tailored sets with a small number of eigenvectors which, though not suitable for general-purposes studies, could be useful for experimental profiling when restricted to a small subset of relevant processes. These and related issues will be the subject of future work.

## Figures and Tables

**Fig. 16:**
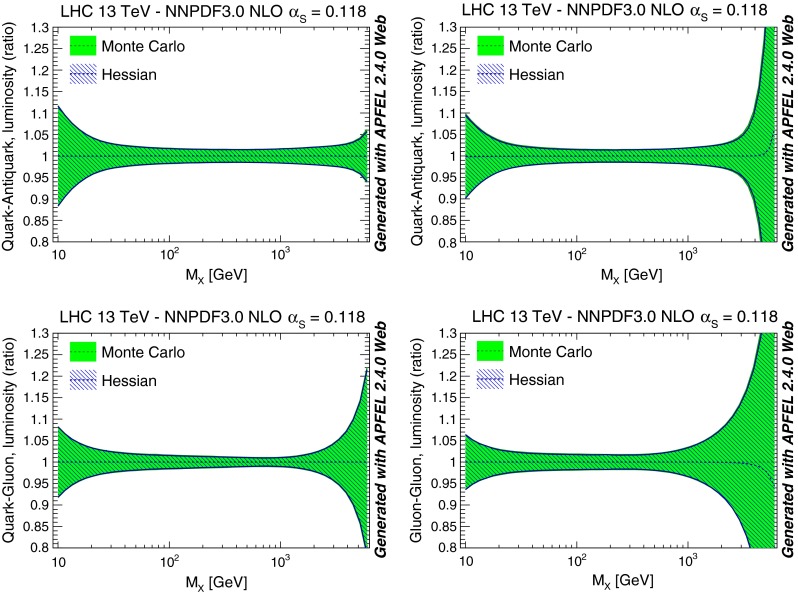
Same as Fig. 8 but now with the Hessian representation constructed using the method discussed in this Appendix

**Fig. 17:**
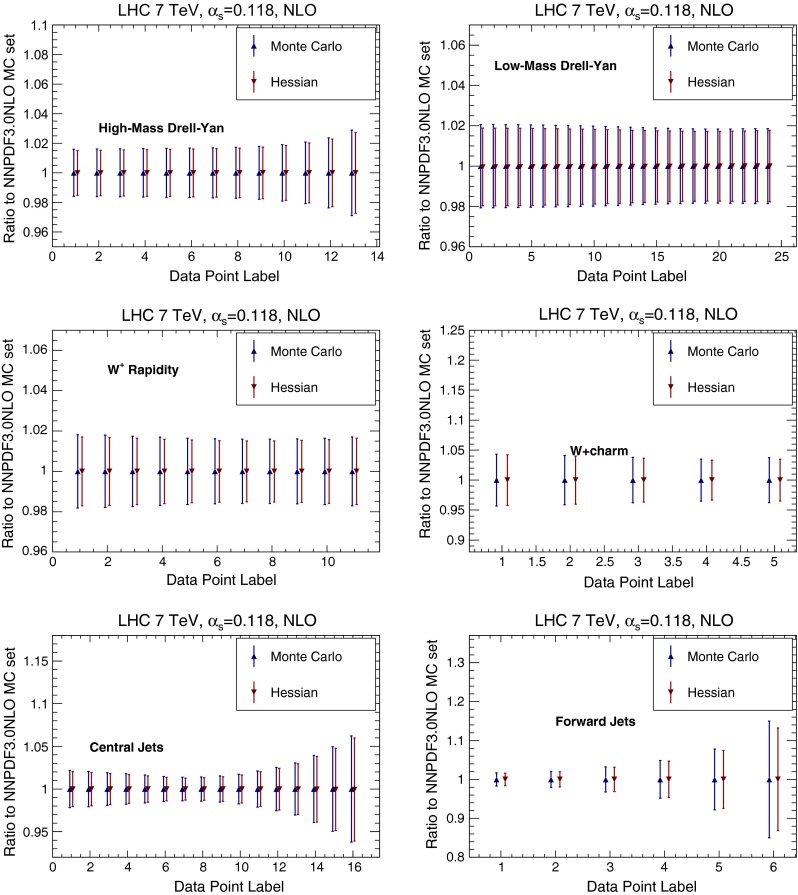
Same as Fig. 12 but now with the Hessian representation constructed using the method discussed in this Appendix

## Appendix A: Hessian representation through Singular Value Decomposition

The solution to the problem of finding a suitable Hessian representation of MC PDF sets is not unique. The main strength of the approach we have explored here is that a representation in terms of the original MC replicas automatically inherits a number of useful properties of the replicas, such as, for instance, the fact that sum rules are automatically satisfied and correlations are well reproduced, as discussed in Sect. 3.1. This suggests an alternative approach, in which instead of representing all starting replicas on a subset of them, we pick the dominant combination of all replicas through Singular Value Decomposition (SVD). This alternative method is briefly discussed in this Appendix. So far we have used it for validation of our main methodology, though it might be especially suitable for future applications, such as the construction of reduced eigenvector sets for specific physical processes.

Since the replicas of a MC set are continuous functions with finite correlation length, the general problem of finding a Hessian representation of a MC set can be interpreted as that of finding a representation of a discrete covariance matrix of the form Eq. (2): the sampling in the space of *x* needs only to be fine grained enough that differences between neighboring points are non-negligible.

Such representation can be directly constructed in terms of Monte Carlo replicas, in the following way. We define the rectangular $(N_{x}N_{f})\times N_{\mathrm{{rep}}}$ matrix

16 $$\begin{aligned} X_{lk}= f^{(k)}_{\alpha }(x_i,Q_0) - f^{(0)}_{\alpha }(x_i,Q_0), \end{aligned}$$

where we adopted the same convention for indices as in Eq. (5): $\alpha$ labels PDFs, *i* points in the *x* grid, $l \equiv N_x(\alpha -1) + i$ runs over all $N_{x}N_{f}$ grid points, and *k* runs over all MC replicas. The covariance matrix Eq. (2) is equal to

17 $$\begin{aligned} \mathrm{{cov}}^{\mathrm{{pdf}}}_{ij,\alpha \beta }= {{1}\over {N_{\mathrm{{rep}}} - 1}} XX^t. \end{aligned}$$

A diagonal representation of the covariance matrix in terms of replicas is found by SVD of the matrix *X*. Namely, we can write *X* as:

18 $$\begin{aligned} X=USV^t. \end{aligned}$$

Assuming that $N_{\mathrm{{pdf}}}N_x<N_{\mathrm{{rep}}}$, *U* is an orthogonal matrix of dimensions $N_{\mathrm{{pdf}}}N_x\times N_{\mathrm{{rep}}}$ which contains the orthogonal eigenvectors of the covariance matrix with nonzero eigenvalues; *S* is a diagonal matrix of real positive elements, constructed out of the singular values of *X*, i.e. the square roots of the nonzero eigenvalues of $\mathrm{{cov}}^{\mathrm{{pdf}}}$ multiplied by the normalization constant $(N_{\mathrm{{rep}}} - 1)^{{1}\over {2}}$; and *V* is an orthogonal $N_{\mathrm{{rep}}} \times N_{\mathrm{{rep}}}$ matrix of coefficients.

Because

19 $$\begin{aligned} XX^t = US^2U^t = (US)(US)^t, \end{aligned}$$

the matrix

20 $$\begin{aligned} Z= US \end{aligned}$$

has the property that

21 $$\begin{aligned} ZZ^t= XX^t. \end{aligned}$$

But also,

22 $$\begin{aligned} Z= XV \end{aligned}$$

and thus *Z* provides the sought-for representation of the covariance matrix as a linear combination of MC replicas.

We have thus arrived at an exact Hessian representation of the covariance matrix in terms of replicas, but with the disadvantage that the number of Hessian eigenvectors parameters is now equal to $N^{(0)}_{\mathrm{{eig}}}=N_{\mathrm{{pdf}}}N_x$, which is generally large. However, in practice many of these eigenvectors will lead to a very small contribution to the covariance matrix. We can then select a smaller set of $N_{\mathrm{{eig}}}<N^{(0)}_{\mathrm{{eig}}}$ eigenvectors which still provides a good approximation to the covariance matrix.

A possible strategy is, for example, to select the $N_{\mathrm{{eig}}}$ eigenvectors with largest singular values. Denoting with *u*, *s*, and *v* the $N_{\mathrm{{pdf}}}N_x\times N_{\mathrm{{eig}}}$, $N_{\mathrm{{eig}}}\times N_{\mathrm{{eig}}}$ and $N_{\mathrm{{eig}}} \times N_{\mathrm{{rep}}}$ reduced matrices computed using these eigenvalues, for a given value of $N_{\mathrm{{eig}}}$, using *v* instead of *V* in Eq. (22) minimizes the difference between the original and reduced covariance matrix

23 $$\begin{aligned} \Delta \equiv \Vert US^2U^t - us^2u^t\Vert . \end{aligned}$$

We have verified that the method described in this Appendix provides comparable results to those of the main strategy discussed in the paper. To this purpose, we have selected the set of $N_{\mathrm{{eig}}}=120$ eigenvectors that minimize the figure of merit Eq. (23). To illustrate the quality of the new method in Fig. 16 we show a comparison of PDF luminosities, analogous to Fig. 8, and in Fig. 17 a comparison of LHC differential distributions, analogous to Fig. 12, but now obtained with the new method. Is clear that the agreement is comparable as that found with the previous method.

The main shortcoming of this method is that minimizing Eq. (23) is not necessarily the best strategy for the construction of an optimal general-purpose eigenvector set, in that it does not always lead to also minimizing the figure of merit Eq. (13) which was shown in Sect. 2.2 to be physically advantageous. Some tuning of the methodology would thus be required.

## Footnotes

1 Note that the CT14 PDF set included in this combination is still preliminary. We thank Pavel Nadolsky for providing this preliminary version of CT14.
